# Strategy to enhance transgene expression in proximity of amyloid plaques in a mouse model of Alzheimer's disease

**DOI:** 10.7150/thno.36718

**Published:** 2019-10-18

**Authors:** Danielle Weber-Adrian, Rikke Hahn Kofoed, Josephine Wing Yee Chan, Joseph Silburt, Zeinab Noroozian, Sebastian Kügler, Kullervo Hynynen, Isabelle Aubert

**Affiliations:** 1 Biological Sciences, Hurvitz Brain Sciences Research Program, Sunnybrook Research Institute, Toronto, ON, Canada; 2 Department of Laboratory Medicine and Pathobiology, University of Toronto, Toronto, ON Canada; 3 Department of Neurology, Georg-August-Universität Göttingen, Göttingen, Germany; 4 Physical Sciences, Sunnybrook Research Institute, Toronto, ON, Canada; 5 Department of Medical Biophysics, University of Toronto, Toronto, ON, Canada

**Keywords:** focused ultrasound, gene expression, TgCRND8 mice, astrocytes, amyloid-beta peptides

## Abstract

Gene therapy can be designed to efficiently counter pathological features characteristic of neurodegenerative disorders. Here, we took advantage of the glial fibrillary acidic protein (GFAP) promoter to preferentially enhance transgene expression near plaques composed of amyloid-beta peptides (Aβ), a hallmark of Alzheimer's disease (AD), in the TgCRND8 mouse model of amyloidosis.

**Methods:** The delivery of intravenously injected recombinant adeno-associated virus mosaic serotype 1/2 (rAAV1/2) to the cortex and hippocampus of TgCRND8 mice was facilitated using transcranial MRI-guided focused ultrasound in combination with microbubbles (MRIgFUS), which transiently and locally increases the permeability of the blood-brain barrier (BBB). rAAV1/2 expression of the reporter green fluorescent protein (GFP) under a GFAP promoter was compared to GFP expression driven by the constitutive human beta actin (HBA) promoter.

**Results:** MRIgFUS targeting the cortex and hippocampus facilitated the entry of rAAV1/2 and GFP expression under the GFAP promoter was localized to GFAP-positive astrocytes. Adjacent to Aβ plaques where GFAP is upregulated, the volume, surface area, and fluorescence intensity of the transgene GFP were greater in rAAV1/2-GFAP-GFP compared to rAAV1/2-HBA-GFP treated animals. In peripheral organs, GFP expression was particularly strong in the liver, irrespective of the promoter.

**Conclusion:** The GFAP promoter enhanced transgene expression in proximity of Aβ plaques in the brain of TgCRND8 mice, and it also resulted in significant expression in the liver. Future gene therapies for neurological disorders could benefit from using a GFAP promoter to regulate transgene expression in response to disease-induced astrocytic reactivity.

## Introduction

Recent successes in gene therapy clinical trials include improvements in the vision of patients with leber congenital amaurosis 1, and the first life-saving treatment of neurodegeneration in infants with spinal muscular atrophy 2. These breakthroughs and the advancement of recombinant adeno-associated viruses (rAAVs) have renewed interest in gene therapy for neurological disorders 3-5. However, for most disorders of the central nervous system (CNS), challenges in translating gene therapy approaches to the clinic include delivery across the blood-brain barrier (BBB) 6,7, and the control of transgene expression 8. Though some more recent rAAVs, such as the AAV9 variant AAV-PHP.B, have been shown to overcome the BBB, they cannot be targeted to regions within the brain after systemic delivery 9, which could increase the risk of off-target effects 9. Additionally, the increased brain bioavailability of some of these new capsid variants may be unique to rodents and not observed in non-human primates 10,11 compared to rAAV9.

Alternatively, MRI-guided focused ultrasound combined with microbubbles (MRIgFUS) can be used to transiently and locally disrupt the BBB and the blood-spinal cord barrier to deliver non-BBB penetrating rAAVs, or rAAVs at lower systemic doses, from the bloodstream to targeted regions of the brain and spinal cord 12-19. Recently, ultrasound-mediated BBB permeability has entered clinical trials to establish the safety of the procedure in patients with Alzheimer's disease (AD) 20. When compared to intracranial injections, MRIgFUS delivery of therapeutics to the brain is less invasive, thereby mitigating risks associated with surgical procedures, including infection 21 and tissue damage 22. Additionally, a single MRIgFUS session can cover several areas of the brain or spinal cord with multiple focal points. Intraparenchymal injection of rAAV is associated with limited diffusion and coverage. For example, the cross sectional area of both human hippocampi would require an impractical amount (>50) of intracranial injections 23-26.

In terms of control following systemic injection, cell-specific promoters can modulate transgene expression in the CNS and in peripheral organs. To that end, the astrocyte-associated, 2.2 kilobase pair (kbp) glial fibrillary acidic protein (GFAP) promoter 27 was tested to control rAAV-mediated green fluorescent protein (GFP) expression. In AD brains where amyloid-beta peptides (Aβ) are present, astrocytes in proximity to plaques and throughout the neuropil contribute to the observed increase in endogenous GFAP immunoreactivity 28. As of three months of age, the TgCRND8 mice demonstrate Aβ deposition in the cortex and hippocampus 29. They likewise demonstrate an increase in astrogliosis measured by GFAP starting at three and half months of age, which progresses with age and Aβ pathology 30. Here, the cortex and hippocampus were targeted with MRIgFUS, in the presence of microbubbles, to facilitate BBB delivery of rAAV1/2-GFP under control of either the GFAP promoter or the constitutive human beta actin (HBA) promoter. GFP expression under the GFAP promoter was significantly higher with respect to fluorescence intensity, as well as volume and surface area of transgene protein distribution in GFAP-positive cells (astrocytes) associated with Aβ plaque, compared to non-Aβ affiliated astrocytes, or astrocytes transduced with rAAV-GFP under control of the HBA promoter. The GFAP promoter permits Aβ-responsive expression, resulting in targeted increases in transgene expression corresponding to increases in Aβ-mediated astrocytic activation. Thus, this expression system could provide a form of therapeutic transgene control that self-modulates with disease progression.

## Results

### MRIgFUS facilitates targeted rAAV1/2 delivery to the cortex and hippocampus

**Figure 1:**
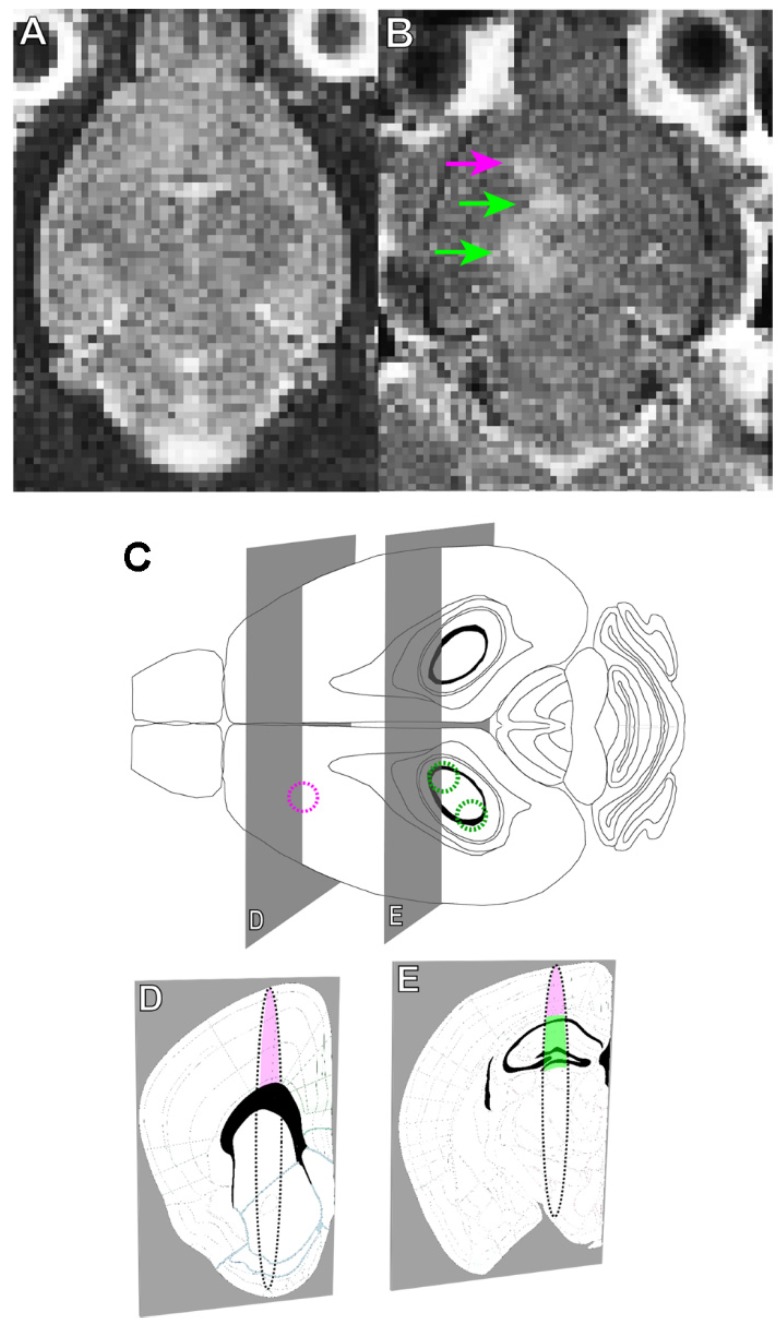
** MRI-guided focused ultrasound (MRIgFUS) mediates blood-brain barrier (BBB) opening in the cortex and hippocampus. (**A) A T2-weighted MRI image is used to target brain regions with focused ultrasound (FUS). **(B)** BBB opening was verified using gadolinium enhancement (arrows: purple targeting the cortex, green targeting the hippocampus), as seen on the T1-weighted MRI image acquired immediately after FUS. **(C)** FUS was applied using one focal point targeting the cortex (purple circle) and two focal points targeting the hippocampus (green circles). (D and E). The focal spot generated using these parameters is oval in shape, which is demonstrated in the coronal perspective. The focal spots include regions of the cortex (pink) and hippocampus (green), which contain deposits of Aβ plaque in TgCRND8 mice as of 3 months of age. **(D** and **E)** Brain atlas images were adapted from the Allen Mouse Brain Atlas.

**Figure 2:**
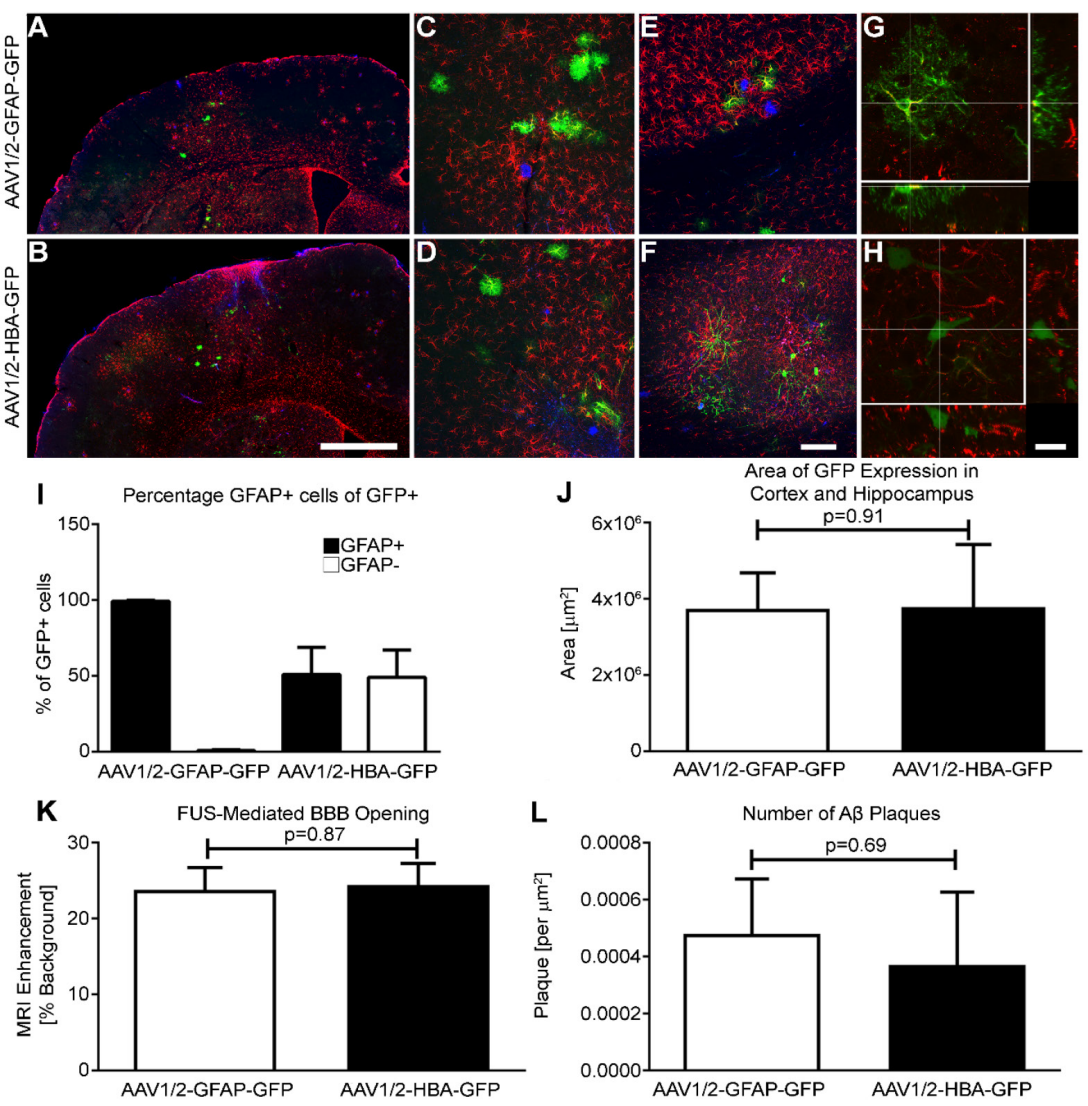
** The GFAP promoter is astrocyte-specific. (A and B)** Two weeks after systemic delivery of either rAAV1/2-GFAP-GFP or rAAV1/2-HBA-GFP, transgene-positive (GFP, green) cells are visible in the FUS-targeted region. Aβ plaque is shown in blue, and GFAP expression in red. **(C)** At higher magnification in the cortex, the morphology of GFP-positive cells in the rAAV1/2-GFAP-GFP group is consistent and colocalizes with GFAP-positive cells (colocalization, yellow). **(D)** In the cortex after delivery of rAAV1/2-HBA-GFP, a variety of cell morphologies are visible and are not always colocalized with GFAP. **(E and F)** In the hippocampus, the same respective trends are seen of GFP-positive cell morphology after delivery of rAAV1/2-GFAP-GFP or rAAV1/2-HBA-GFP. **(G)** As seen in an orthogonal projection, consistent colocalization between GFP-positive cells and GFAP is verified in the rAAV1/2-GFAP-GFP group. **(H)** A consistent colocalization between GFP and GFAP is not seen in the orthogonal projection of rAAV1/2-HBA-GFP cells. **(I)** Quantification of GFP-positive (GFP+) cells are categorized as GFAP+ (astrocyte), or GFAP- (undefined). The percentage of GFP-positive cells that are also GFAP-positive after rAAV1/2-GFAP-GFP delivery indicate almost exclusive transgene expression in astrocytes. **(J)** The areas containing GFP-positive cells in the cortex and hippocampus after FUS application were not significantly different in size (µm^2^) between the rAAV1/2-GFAP-GFP and rAAV1/2-HBA-GFP groups (p=0.91). **(K)** The amount of BBB opening by FUS application can be estimated by the MRI enhancement from background of each focal spot, which was not significantly different between the two rAAV groups (p=0.87). **(L)** The number of Aβ plaques in the cortical and hippocampal regions containing GFP-positive cells was also not significantly different between rAAV groups (p=0.69). Data is represented as mean ±SEM and **(I-L)** n=4 animals per group. **(K)** For MRI enhancement n=12 focal spots from 4 animals, per group. Scale bars: **(A and B)** 1 mm; (c-f) 100 µm; **(G and H)** 20 µm.

Briefly, rAAV1/2-GFAP-GFP or rAAV1/2-HBA-GFP were injected at a dose of 3 x 10^9^ vector genomes per gram (VG/g) through a tail vein catheter in TgCRND8 mice. FUS application immediately preceded viral injection, for which the mice were placed in dorsal recumbency over a spherical ultrasound transducer, as previously described 31. MRI images were used to target FUS to the cortex and hippocampus, and contrast-enhanced MRI was used to verify BBB opening and location (Figure 1A, B). Three, non-overlapping spots were used to target the cortex (1 spot) and hippocampus (2 spots) in each animal (Figure 1C, D, E). Results show that GFP expression from the rAAV1/2-GFAP-GFP or rAAV1/2-HBA-GFP constructs were concentrated and limited to the FUS-targeted regions 14 days post-delivery (Figure 2A, B). Following delivery of rAAV1/2-GFAP-GFP, GFP-expressing cells in the cortex (Figure 2C) and hippocampus (Figure 2E) also expressed GFAP (Figure 2G), indicating astrocyte specificity of the GFAP promoter. GFP-expressing cells in the cortex (Figure 2D) and hippocampus (Figure 2F) of the rAAV1/2-HBA-GFP group did not always co-localize with GFAP (Figure 2H), as the HBA promoter allows for transgene expression in a variety of cell types 32-34.

### GFAP and HBA promoters result in comparable numbers of GFP-positive cells

Firstly, the percentage of GFAP-positive cells within the population of cells expressing GFP was quantified in both the rAAV1/2-GFAP-GFP and rAAV1/2-HBA-GFP groups (Figure 2I). This confirmed that the GFAP promoter leads to preferential transgene expression in astrocytes.

After MRIgFUS delivery, the areas of GFP-expression within the cortex and hippocampus of the rAAV1/2-GFAP-GFP and rAAV1/2-HBA-GFP groups were not significantly different (p=0.91) (Figure 2J), indicating that the focal spots were able to mediate permeabilization of a consistent size. Furthermore, the amount of BBB opening by FUS, as measured by the MRI enhancement from background also confirms that delivery of the rAAV1/2-GFAP-GFP and rAAV1/2-HBA-GFP groups was not significantly different (p=0.87) (Figure 2K). As an additional confirmation of baseline consistency, the average number of Aβ plaques within the FUS-targeted regions was compared between viral groups and was not significantly different (p=0.69) (Figure 2L). Collectively, this supports that differences in transgene expression are due to the GFAP versus HBA promoter transcriptional control, and not to differences in FUS delivery or plaque load within the targeted areas.

### GFP expression under the GFAP promoter is enhanced near Aβ plaque

This study was designed to characterize and quantify the possible increase in transgene expression near Aβ plaque under control of the GFAP promoter, compared to expression unassociated with Aβ plaque, or under control of a constitutive promoter.

**Figure 3:**
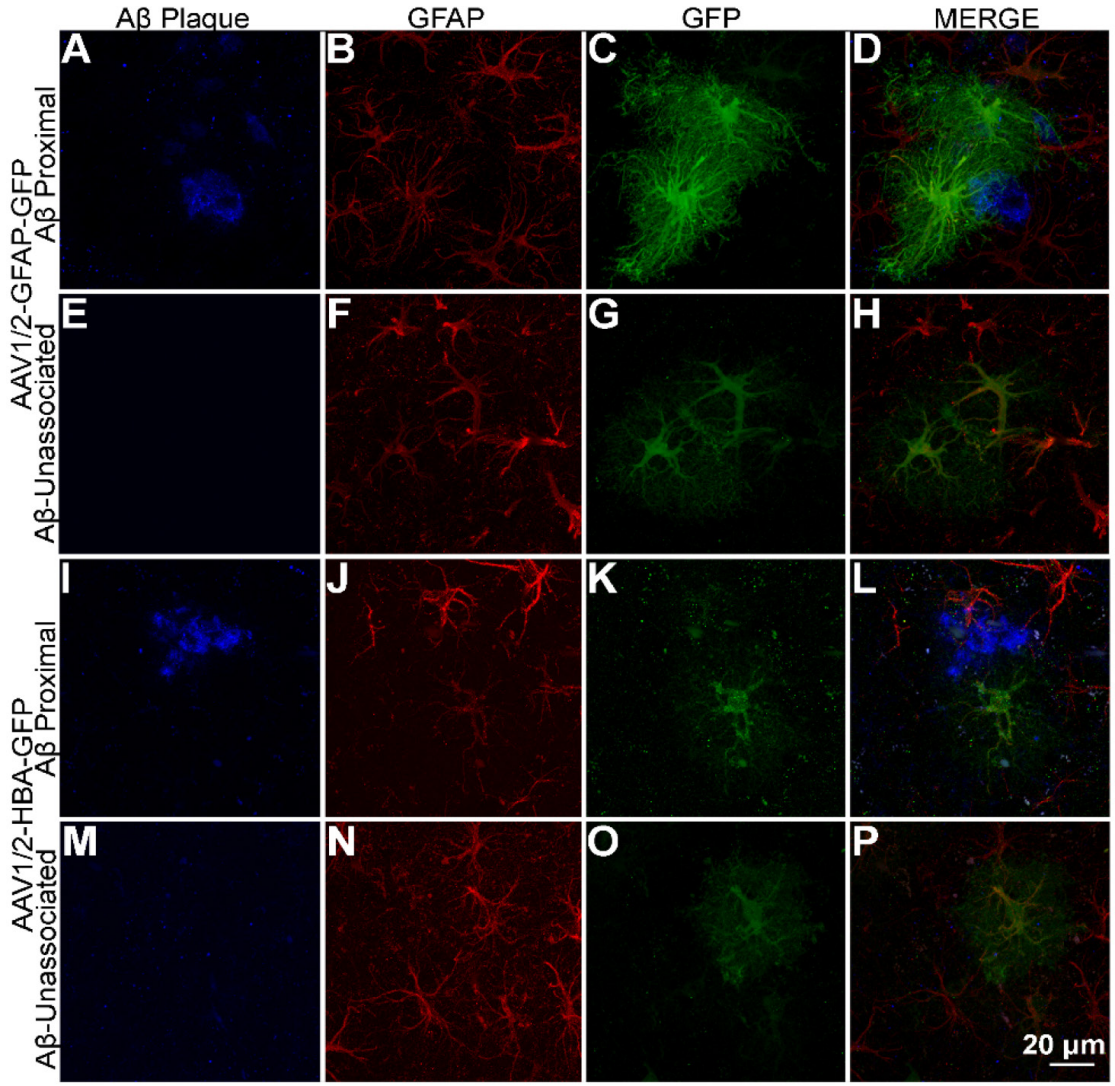
** GFAP promoter-driven expression of GFP intensified in the vicinity of Aβ plaque. (A-D)** rAAV1/2-GFAP-GFP expression in GFAP-positive cells (red) with processes overlapping in space with Aβ plaque (blue) show a distinct pattern of expression in both the cell body and processes compared to **(E-H)** rAAV1/2-GFAP-GFP expression in the absence of Aβ plaque and rAAV1/2-HBA-GFP expression both **(I-L)** associated and **(M-P)** unassociated with Aβ plaque. Scale bar, 20 µm.

Among the astrocytes (GFAP-positive cells) where GFP is expressed under the GFAP promoter, GFP expression shows a distinct distribution pattern in astrocytes with processes overlapping Aβ plaque (Figure 3A-D), compared to astrocytes unassociated with Aβ plaque (Figure 3E-H). On the other hand, GFP expression under the HBA promoter do not show a visible difference in GFP distribution pattern in astrocytes with processes overlapping Aβ plaque (Figure 3I-L), compared to astrocytes unassociated with Aβ plaque (Figure 3M-P). This suggests that expression of GFP under the GFAP promoter but not the HBA is affected by Aβ pathology. The observed promoter- differences in GFP distribution within Aβ-associated astrocytes were not caused by differences in cell morphology (Figure 3B and J).

**Figure 4:**
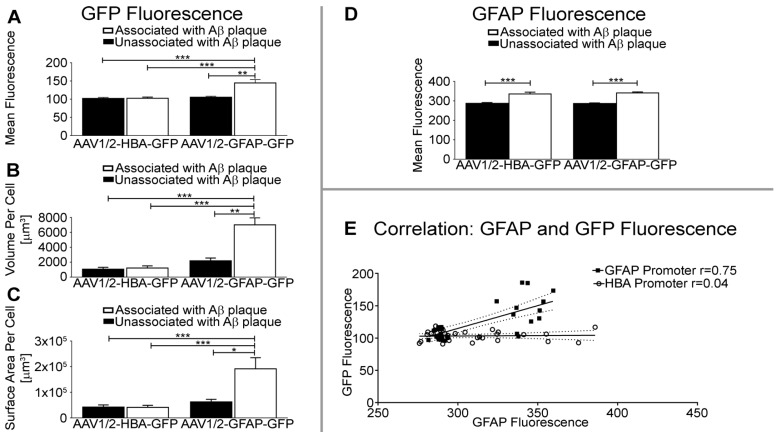
** GFAP promoter results in greater GFP fluorescence intensity, volume, and surface area of astrocytes near Aβ plaque. (A)** Quantification of GFP fluorescence per unit volume is significantly increased in GFAP and GFP-positive cells near Aβ plaque in the rAAV1/2-GFAP-GFP group, compared to GFAP and GFP-positive cells unassociated with Aβ plaque (**p<0.01), or GFAP and GFP-positive cells from the rAAV1/2-HBA-GFP group (***p<0.001). **(B)** The volume of GFP distribution per GFAP-positive cell was significantly higher in the rAAV1/2-GFAP-GFP group near Aβ plaque, compared to GFP expression isolated from Aβ (**p<0.01), or compared to GFP-positive cells of the rAAV1/2-HBA-GFP group (***p<0.001). **(C)** Surface area of GFP distribution was also significantly greater in GFP and GFAP-positive cells proximal to Aβ plaque in the rAAV1/2-GFAP-GFP group, compared to cells unassociated with Aβ (*p<0.05), or GFP and GFAP-positive cells from the rAAV1/2-HBA-GFP group (***p<0.001). **(D)** Quantification of GFAP fluorescence per unit volume is significantly increased in GFP-positive cells associated with plaques compared to unassociated in both the rAAV1/2-GFAP-GFP and rAAV1/2-HBA-GFP groups (***p<0.001). **(E)** GFP (transgene) fluorescence intensity under control of the GFAP promoter (black squares) is correlated (r=0.75 p<0.0001, solid line represents line of best fit, dotted lines show 95% confidence interval) with GFAP protein fluorescence in the same cell, while transgene expression under the HBA promoter (open circles) was not correlated with GFAP protein fluorescence intensity (r=0.04 p=0.81). Data is represented as mean ± SEM. For each rAAV group, n=12 z-stack images were used to create 3D representations of GFP and GFAP-positive cells near Aβ plaque, and n=20 z-stack images were used for GFP and GFAP-positive cells unassociated with Aβ plaque.

Quantification of mean fluorescence intensity per unit volume shows that GFP expression under the GFAP promoter in astrocytes is significantly higher near Aβ plaque than in astrocytes unassociated with Aβ (p<0.01), or under control of the HBA promoter (Aβ-associated, p<0.001; Aβ-unassociated, p<0.001) (Figure 4A). The volume of GFP distribution was also significantly higher in astrocytes associated with Aβ plaque relative to astrocytes unassociated with Aβ in the rAAV1/2-GFAP-GFP group (p<0.01), and compared to all GFP-positive astrocytes from the rAAV1/2-HBA-GFP group (p<0.0001) (Figure 4B). Additionally, the surface area of GFP distribution was significantly greater in astrocytes associated with Aβ plaque in the rAAV1/2-GFAP-GFP compared to astrocytes unassociated with Aβ (p<0.05), and to GFP-positive astrocytes from the rAAV1/2-HBA-GFP group (p<0.001) (Figure 4C). Fluorescence intensity, volume, and surface area were not significantly different between non-Aβ associated, GFP-positive astrocytes under control of the GFAP promoter, and GFP-positive astrocytes under control of the HBA promoter (p>0.05).

Transgene expression (GFP), under the control of the GFAP promoter, was increased by 37% in astrocytes associated with Aβ plaques, compared to non-Aβ associated astrocytes (rAAV1/2-GFAP-GFP, Figure 4A). This 37% increase in GFP expression is visually evident (Figure 3). In contrast, GFAP fluorescence in Aβ plaque-associated astrocytes positive for GFP is not always noticeably increased qualitatively, although it was significantly increased by 19% and 17% in the rAAV1/2-GFAP-GFP and rAAV1/2-HBA-GFP groups, respectively (Figure 4D).

GFP and GFAP fluorescence intensities in GFP-positive astrocytes strongly correlated in the rAAV1/2-GFAP-GFP group (r=0.75, p<0.0001), but not in the rAAV1/2- HBA-GFP group (r=0.04, p=0.81) (Figure 4E). This supports that the increase in GFP near plaques are due to an increase in GFAP promoter activity.

### GFAP promoter limits transgene expression in periphery, with exception of the liver and kidney

**Figure 5:**
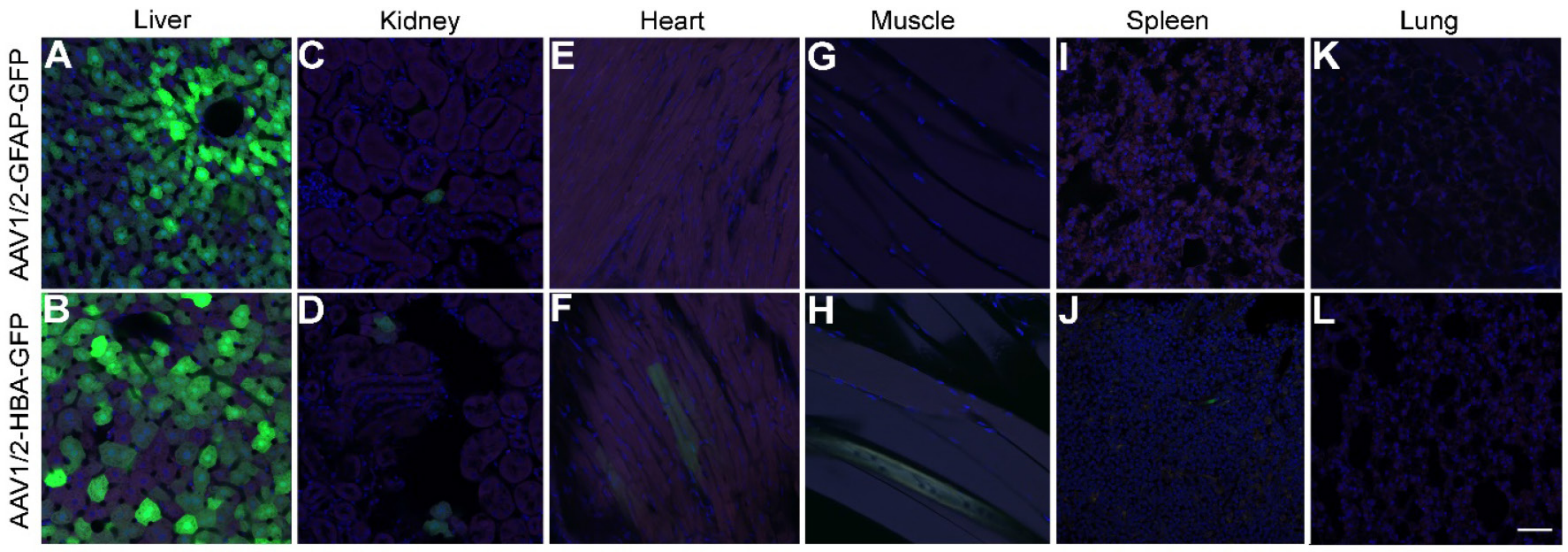
** GFAP promoter permits transgene expression in the liver. (A)** Under control of the GFAP promoter, GFP expression (green) was not prevented in the liver, despite an absence of GFAP protein detection (red). Cell nuclei are shown in blue (DAPI). **(B)** Under control of the HBA promoter, GFP was also expressed in the liver. **(C and D)** Both promoters lead to expression in the kidney, **(E and F)** while only the rAAV1/2-HBA-GFP group showed GFP expression in the heart. **(G)** GFP expression was not seen in the quadriceps muscle of the rAAV1/2-GFAP-GFP group, **(H)** but was detected in the rAAV1/2-HBA-GFP group. **(I)** GFP expression was not detected in the spleen after delivery of rAAV1/2-GFAP-GFP, **(J)** but was detected in the rAAV1/2-HBA-GFP group. **(K and L)** No GFP expression was detected in the lung for either the GFAP or HBA promoter groups. **(A-L)** Scale bar, 50 µm.

Although expression of rAAV1/2-GFP under the GFAP promoter in the cortex and hippocampus led to almost exclusive colocalization with GFAP-positive cells (Figure 2I), this was not the case in the liver (Figure 5A) where GFP expression was seen, even in the absence of detectable GFAP expression. GFP expression was also visible in the liver after delivery of rAAV1/2-HBA-GFP (Figure 5B). A few GFP positive cells were seen in the kidney in both the GFAP promoter group (Figure 5C) and in the HBA promoter group (Figure 5D); GFP expression under control of the GFAP promoter was not detected in the heart (Figure 5E), muscle (Figure 5G), spleen (Figure 5I), or lung (Figure 5K). In contrast, a few GFP positive cells were seen when rAAV1/2-GFP was expressed under the HBA promoter in the heart (Figure 5F), muscle (Figure 5H), and spleen (Figure 5J), but no positive cells were detected in the lung (Figure 5L).

## Discussion

Using a GFAP promoter, we have demonstrated that transgene expression (i.e. GFP) is increased alongside GFAP-positive areas of astrocytic activation. This finding introduces the possibilities of augmenting therapeutic delivery near pathological hallmarks and regulating transgene expression in response to disease progression and therapeutic effects. GFAP expression is increased in several disorders and injuries of the central nervous system including AD 35,36, amyotrophic lateral sclerosis 37, and multiple system atrophy 38, as well as rodent models of traumatic brain injury 39,40. In a mouse model of AD, Vitale et al. found increased efficacy in reducing tau pathology when using a GFAP promoter to express anti-tau antibodies, compared to an ubiquitous promoter 41. Here, enhanced transgene expression near astrogliosis is exemplified using Aβ plaque deposition in the TgCRND8 mouse model of amyloidosis.

rAAV1/2-GFAP-GFP and rAAV1/2-HBA-GFP were delivered from the bloodstream of TgCRND8 mice to the cortex and hippocampus using MRIgFUS. GFP expression under the GFAP promoter led to heightened GFP fluorescence intensity and increased GFP distribution volume and surface area in astrocytes near Aβ plaque, compared to GFP-positive astrocytes unassociated with Aβ plaque, and GFP-positive astrocytes from the rAAV1/2-HBA-GFP delivery group. This demonstrates that transgenic protein quantity and distribution throughout astrocyte processes can be selectively enhanced in the vicinity of Aβ, thereby increasing therapeutic delivery alongside plaque load. Additionally, FUS has been shown to increase GFAP expression at four, and up to fifteen, days following application in the targeted cortex of TgCRND8 mice 42. Together with the results shown here, this suggests that the GFAP promoter can be utilised to both increase transgene expression near Aβ plaques as well as to boost transgene expression in all transduced cells by reapplication of FUS. Gene therapy is traditionally a one-chance treatment which often does not allow for retreatments due to expression of anti-AAV antibodies following the first administration. Future studies will investigate the GFAP promoter as a means to enable a boosting of therapeutic expression by reapplication of FUS to increase GFAP promoter activity.

The increase in endogenous GFAP expression in association with Aβ plaques is approximately half that of GFP expressed under the GFAP promoter. Discrepancies between endogenous GFAP and GFP expressed under a GFAP promoter have been previously reported, and suggested to be caused by the different subcellular localizations of the GFAP and GFP proteins 43. Others have also shown that increased GFAP in TgCRND8 mice correlates with age and Aβ pathology, but that it is also variable and not only found near thioflavin-positive plaques 30,44,45. Regardless of these fluorescence discrepancies, GFP demonstrated a correlation with GFAP fluorescence when under the control of the GFAP promoter, suggesting their regulation is strongly linked.

Protein expression has been correlated *in vitro* 46, in E. coli 47, and in mice 48 with transgene fluorescence intensity. However, a caveat to using fluorescence intensity to measure protein expression is that its precision is vulnerable to changes in fluorescence background 49, self-aggregation of fluorescent species 50, regional differences in pH 51, photobleaching 51, and pixel saturation 49,52. In order to compliment GFP quantification in a manner that was independent of differences in regional intensity, the volume and surface area of GFP distribution within GFAP-positive cells were also measured. Although volume provides a 3D measurement of space occupied by GFP expression, surface area is more sensitive to distribution within astrocyte processes 53.

In the currently described findings, GFP expression under control of the GFAP promoter was found in the liver, and to a limited extent in the kidney; however, endogenous GFAP expression was not detected (Figure 5). Previous studies have shown variable results in transgene expression in the liver under control of the same 2.2 kbp GFAP promoter (gfp2) used here, with some results showing transgene expression in the liver 54,55 and others finding no detectable transgenic protein 43,56. The presence of transgene expression under the GFAP promoter in the absence of GFAP could be related to access restriction of highly condensed chromatin containing the genomic GFAP promoter and gene sequence, or variable trans and cis chromosomal interactions, which may not affect transgene expression from an episomal GFAP promoter construct 57. It is also known that hepatic stellate cells representing 5-8% of the human liver cells express GFAP 58. On the other hand, a point of consideration for transgene expression in the liver is duration, as rAAV-mediated transgene expression can be lost after several weeks, whereas expression in the brain has been shown to persist for several years 59,60. The mechanisms of expression loss in the liver 17, and inconsistencies in GFAP promoter-driven expression within off-target organs 43,54-56 remain to be fully elucidated. In order to prevent even transient expression in the liver after systemic delivery of rAAV1/2-GFAP, future investigations could utilize organ-specific microRNA inhibition 61.

## Conclusion

Our results provide proof-of-concept for a novel approach using MRIgFUS to facilitate non-surgical delivery of a gene vector containing a GFAP promoter, hereby enhancing transgene expression in astrocytes surrounding amyloid plaques. In GFP-positive astrocytes associated with Aβ plaque in the rAAV1/2-GFAP-GFP group, fluorescence intensity, as well as volume and surface area of GFP distribution was increased, compared to astrocytes unassociated with Aβ plaque, or transgene-positive astrocytes from the rAAV1/2-HBA-GFP delivery group. This data illustrates the potential of the GFAP promoter to target and increase transgene expression alongside astrocyte activation and pathology, and future studies will evaluate the efficacy of therapeutic molecules expressed under the control of a GFAP promoter to decrease Aβ pathology. Provided that astrogliosis occurs in cases of neurodegeneration, neuroinflammation, stroke and other types of injuries of the central nervous, the use of promoters responding to astrogliosis could be beneficial in curbing disease progression.

## Methods

### Animals

TgCRND8 mice were used at 15 weeks of age, with an average mass of 26 grams. The animal procedures carried out in these experiments complied with the Canadian Council on Animal Care and the Animals for Research Act of Ontario guidelines, and were approved by the Sunnybrook Research Institute Animal Care Committee. The rAAV1/2-GFAP-GFP and rAAV1/2-HBA-GFP delivery groups each included four mice.

### Virus Preparation

rAAV1/2 expressing enhanced GFP was generated under control of either the 2,210 base pair human GFAP promoter, or the HBA promoter as previously described 27,33. Briefly, rAAV1 and rAAV2 packaging plasmids were used at a 50:50 ratio to generate mosaic rAAV1/2 particles, which were purified using iodixanol gradient centrifugation and fast protein liquid chromatography on heparin affinity columns. To increase expression, the cytomegalovirus enhancer sequence was included directly upstream of the HBA promoter. The woodchuck hepatitis virus posttranscriptional regulatory element was included after the GFP sequence to enhance mRNA stability, along with the bovine growth hormone polyadenylation sequence. rAAV virus was injected at a dose of 3 x 10^9^ VG/g through a 22-G angiocatheter in the tail vein for MRIgFUS delivery.

### Magnetic Resonance Imaging-Guided Focused Ultrasound (MRIgFUS)

Isofluorane inhalation was used to anesthetize the mice, and depilatory cream was applied to remove hair from the head and neck. The mice were positioned in dorsal recumbency over an MRI radiofrequency surface coil as previously described 31.

A 7T MRI (Bruker BioSpin MRI GmbH, Ettlingen, Germany) was used to generate images of the brain and target regions of the cortex and hippocampus (Figure 1A). Unilateral targeting of the cortex and hippocampus was done using one or two FUS spots, respectively (Figure 1). A 1.68 MHz spherically focused transducer (aperture: 7 cm, F-number: 0.8) was used to generate ultrasound, and was driven using a function generator and radio frequency power amplifier. FUS sonications were applied using 10 msec bursts, at a repetition frequency of 1 Hz, for 120 seconds. To control acoustic pressures, a 4.8 mm diameter wideband polyvinylidene fluoride hydrophone was used as previously described 62. For all sonications, the acoustic pressure amplitude was increased in a step-wise manner, while the hydrophone was used to detect sub-harmonic acoustic emissions. When a 840 kHz sub-harmonic emission was detected by the hydrophone, the pressure amplitude level was dropped to 50% of the value at which the subharmonic had been detected, and maintained for the duration of the sonication. An injection of Definity microbubbles (0.02 ml/kg), followed by saline (200 µL) through the tail vein catheter was given immediately before FUS application. Subsequently, virus was injected (3 x 10^9^ VG/g), followed by saline (200 µL), Gadodiamide MRI contrast agent (0.2 ml/kg, Omniscan, GE Healthcare Canada, Mississauga, ON, Canada), and additional saline (200 µL). Following FUS application, contrast-enhanced T1-weighted MRI images were acquired at a resolution of 0.25 x 0.25 x 1.5 mm in the X x Y x Z axis to visualize the 1 mm^2^ BBB permeability, as demonstrated by regions of enhancement (Figure 1B, arrowheads). Upon recovery from anesthesia, the mice were returned to their cages.

### Tissue Processing

14 days after MRIgFUS application, mice were anesthetized using an intraperitoneal injection of ketamine (75 mg/kg) and xylazine (10 mg/kg). Transcardial perfusion using 0.9% saline and 4% paraformaldehyde solution in 0.1M PO_4_ was performed. The brain and peripheral organs were collected and post-fixed in 4% paraformaldehyde solution for 24 hours, transferred to 30% sucrose solution and then stored at 4°C. The brains and peripheral organs were mounted in Tissue-Tek OCT (Sakura, Torrance, CA, USA), frozen with dry ice and cut in 40 μm-thick sections on a sliding microtome. Sections were kept at -20°C in cryoprotective glycerol solution.

### Immunohistochemistry

Free floating brain sections were rinsed in phosphate-buffered saline (PBS, pH 7.4) for five minutes three times before antigen retrieval, which was done using incubation in 70% formic acid in PBS at room temperature for 5 minutes. The sections were rinsed three times before incubation for 1 hour at room temperature in blocking solution (PBS++) composed of 2% donkey serum (Wisent Bioproducts, Saint-Jean Baptiste, QC, Canada), 1.5% bovine serum albumin (Wisent Bioproducts), and 0.15% Triton X-100 (Sigma-Aldrich Canada, Oakville, ON, Canada) in PBS. Sections were incubated overnight at 4°C in PBS++ containing rabbit anti-GFP (1:500; Millipore, AB3080, Bedford, MA, USA), goat anti-GFAP (1:300, Novus Biologicals, NB100-53809, Littleton, CO, USA) and the anti-Aβ 6F3D antibody (1:200, Dako North American Inc., Carpinteria, CA, USA). Sections were then rinsed again three times in PBS for five minutes and incubated in PBS++ with donkey anti-rabbit biotin (1:200, Jackson ImmunoResearch, 711-065-152, West Grove, PA, USA) for 1 hour at room temperature. After three, five-minute rinses in PBS, sections were incubated in PBS++ with donkey anti-goat Cy3 (1:200; Jackson ImmunoResearch, 705-165-147), donkey anti-mouse Cy 5 (1:200; Jackson ImmunoResearch, 715-175-150), and Alexa Fluor® 488-conjugated streptavidin (1:200, Jackson ImmunoReserach, 016-540-084) for two hours at room temperature. After an additional three five-minute rinses in PBS, the sections were stained with DAPI nucleic acid (1:10,000, Invitrogen, D3571, Eugene, OR, USA) in PBS for 10 minutes, and rinsed before mounting with polyvinyl alcohol (Sigma-Aldrich, St Louis, MO, USA) and 1,4 diazabicyclo(2.2.2)octane (Sigma-Aldrich) (PVA-DABCO) on a microscope slide with a coverslip.

Peripheral organ sections were stained as described above without antigen retrieval in formic acid, and in blocking solution that consisted of 10% donkey serum and 1% TX-100 in PBS. The primary antibodies used were the same rabbit anti-GFP (1:500), donkey anti-GFAP (1:300), and DAPI as described above.

### Imaging

A single mosaic from adjacent 1 μm-step Z-stack images, (Figure 2A and B) taken using a 10x objective (NA 0.5) on an AxioImager M2 (Carl Zeiss, Toronto, ON, Canada), was compiled using 3D Virtual Slice software (Stereo Investigator, MBF Bioscience, Williston, VT, USA). An apochromatically corrected 20x objective (NA 0.75) (Figure 2C-F) and 60x objective (NA 1.4) (Figure 2G and H and Figure 3A-P) on a Nikon A1 laser scanning confocal microscope (Nikon Instruments, Melville, NY, USA) were also used to acquire images. Images are presented as maximum intensity projections from 23, 0.85 μm-step Z-stacks (Figure 2C-F), maximum intensity projections from 88, 0.18 μm-step Z-stacks (Figure 3A-P), or as orthogonal views from 0.1 μm-step Z-stacks (Figure 2G, H).

### Cell Counting

The numbers of Aβ plaques, GFP-positive and GFAP-positive cells, and GFP-positive and GFAP-negative cells within the FUS-targeted areas of the brain were quantified using Stereo Investigator software on a Zeiss AxioImager M2 microscope. GFP expression associated with Aβ plaque was defined as the occurrence of plaque and GFP expression from the GFAP-positive cell body or projections overlapping in space. For coronal brain sections, six 40 μm-thick sections from the FUS-targeted regions were used at an interval of one in six for quantification. For axial brain sections, five 40 µm-thick sections were used at an interval of one in three. Quantification was done using an optical fractionator probe and 63x oil objective (NA= 1.4) on an exhaustive grid covering all regions of the hippocampus and cortex with visible GFP expression. The final number of plaque and cell counts was extrapolated from the section interval. The Cavalieri estimator probe within the Stereo Investigator software was used to estimate area of GFP expression. The mean number of GFP-positive cells, area of GFP expression and number of Aβ plaques were evaluated between the rAAV1/2-GFAP-GFP and rAAV1/2-HBA-GFP experimental groups using a two-tailed, unpaired t-test. The difference in number of GFAP-positive and GFAP-negative cells within the rAAV1/2-GFAP-GFP and rAAV1/2-HBA-GFP groups was evaluated using a two-way ANOVA and Bonferroni post-hoc comparison. A p value of less than 0.05 was considered statistically significant for all analyses. All statistical analyses were performed using GraphPad Prism 5 (GraphPad Software, La Jolla, CA, USA).

### Fluorescence Quantification

The GFP and GFAP fluorescence intensity per unit volume, as well as GFP expression volume and surface area of a GFP and GFAP-positive cell, either associated or unassociated with Aβ plaque, was compared using the 3D Measurement module of Nikon Elements software (Nikon Instruments). This analysis was done using 0.1 µm Z-stack images comprising the entire volume of the GFP-positive cell, taken with a 60x objective (NA 1.4). A sample of 12 Z-stack images of GFP and GFAP-positive cells associated with Aβ plaque was used per rAAV1/2-GFAP-GFP or rAAV1/2-HBA-GFP group. A sample of 20 Z-stack images of GFP and GFAP-positive cells that were unassociated with Aβ plaque were used per rAAV group. The mean fluorescence per unit area, as well as volume and surface area of GFP expression were compared between GFP and GFAP-positive cells either associated or unassociated with Aβ plaque from the rAAV1/2-GFAP-GFP or rAAV1/2-HBA-GFP delivery group using a Kruskal-Wallis one-way analysis of covariance (Gaussian distribution not assumed) and Dunn's multiple comparison test post hoc (GraphPad Prism 5, GraphPad Software).

### MRI Enhancement Quantification

Enhancement at each FUS focal spot was measured from an average of a 3X3 pixel area of the MRI image and expressed as a percentage increase from background enhancement using Matlab (MathWorks, Natick, MA, USA). A two-tailed, unpaired t-test was used to compare the enhancement of all focal spots between the experimental groups (GraphPad Prism 5, GraphPad Software).

## Abbreviations

Aβ: amyloid beta
AD: Alzheimer's disease
BBB: blood-brain barrier
CNS: central nervous system
FUS: focused ultrasound
GFAP: glial fibrillary acidic protein
GFP: green fluorescent protein
HBA: human beta actin
kbp: kilobase pairs
MRI: magnetic resonance imaging
MRIgFUS: MRI-guided focused ultrasound
PBS: phosphate-buffered saline
PVA-DABCO: 1,4 diazabicyclo(2.2.2)octane
rAAV: recombinant adeno-associated virus
VG/g: vector genomes per gram
